# Protein kinase Cε and protein kinase Cθ double-deficient mice have a bleeding diathesis

**DOI:** 10.1111/j.1538-7836.2012.04857.x

**Published:** 2012-09-09

**Authors:** A J UNSWORTH, B A FINNEY, L NAVARRO-NUNEZ, S SEVERIN, S P WATSON, C J PEARS

**Affiliations:** 1 Department of Biochemistry, University of OxfordOxford; 2 Centre for Cardiovascular Sciences, Institute of Biomedical Research, School of Clinical and Experimental Medicine, College of Medical and Dental Sciences, University of BirminghamBirmingham, UK

**Keywords:** aggregation, bleeding times, collagen, fibrinogen, hemostasis, protein kinase C

## Abstract

**Summary:**

**Background:** In comparison to the classical isoforms of protein kinase C (PKC), the novel isoforms are thought to play minor or inhibitory roles in the regulation of platelet activation and thrombosis.

**Objectives:**

To measure the levels of PKCθ and PKCε and to investigate the phenotype of mice deficient in both novel PKC isoforms.

**Methods:**

Tail bleeding and platelet activation assays were monitored in mice and platelets from mice deficient in both PKCθ and PKCε.

**Results:**

PKCε plays a minor role in supporting aggregation and secretion following stimulation of the collagen receptor GPVI in mouse platelets but has no apparent role in spreading on fibrinogen. PKCθ, in contrast, plays a minor role in supporting adhesion and filopodial generation on fibrinogen but has no apparent role in aggregation and secretion induced by GPVI despite being expressed at over 10 times the level of PKCε. Platelets deficient in both novel isoforms have a similar pattern of aggregation downstream of GPVI and spreading on fibrinogen as the single null mutants. Strikingly, a marked reduction in aggregation on collagen under arteriolar shear conditions is observed in blood from the double but not single-deficient mice along with a significant increase in tail bleeding.

**Conclusions:**

These results reveal a greater than additive role for PKCθ and PKCε in supporting platelet activation under shear conditions and demonstrate that, in combination, the two novel PKCs support platelet activation.

## Introduction

Following damage to the blood vessel wall, components of the subendothelial matrix such as collagen become exposed and activate platelets circulating in the blood. Platelet activation involves granule secretion, activation of the major platelet integrin αIIbβ_3_, actin rearrangements and generation of thrombin, which amplifies platelet activation, thrombus formation and hemostasis.

The protein kinase C (PKC) serine/threonine kinase family plays a critical role in the regulation of several processes involved in platelet activation. Broad spectrum inhibition of all PKC family members blocks platelet responses to most agonists, including collagen and thrombin [1–3]. The PKC superfamily consists of 10 isoforms subdivided into classical (α, βI, βII, γ), novel (δ, ε, η, θ) and atypical (ξ, ι/λ) isoforms on the basis of their domain structure and sensitivity to 1,2-diacylglycerol and Ca^2+^. Robust expression of several isoforms has been reported in human (α, β, δ, θ) and mouse (α, β, ε, δ, θ) platelets, with evidence of expression of additional isoforms. In contrast, there are conflicting reports on expression of PKCε in human platelets [4–7], although a recent study using an in-house antibody has demonstrated robust expression [8].

The role of individual isoforms in platelet activation has been investigated using isoform-specific inhibitors and mice deficient in single isoforms [4–7,9–19]. This has led to the conclusion that the classical isoforms play positive roles in platelet activation, with PKCα playing the predominant role, supported by PKCβ, while the novel isoforms play minor or inhibitory roles [10]. There is, however, a need for caution in this generalized overview as there is increasing evidence that individual isoforms of classical and novel PKCs have agonist-specific roles.

This is illustrated by PKCε, which supports activation of mouse platelets by GPVI through serine phosphorylation of the FcR γ-chain, leading to increased binding activation of the tyrosine kinase Syk [6]. In contrast, PKCε has been shown to play a role in the negative regulation of G protein coupled receptor signaling, in particular in the regulation of ADP-induced platelet dense granule secretion [8,20]. In comparison, the novel isoform PKCθ is required for αIIbβ_3_-mediated adhesion and filopodial generation on fibrinogen [14,18] but has only a minor role downstream of GPVI and PAR receptors, with both stimulatory and inhibitory results observed, possibly reflecting subtle changes in experimental conditions [7,10,14,15,18,21,22]. The role of PKCθ in platelet aggregation under flow conditions is unclear, with a stimulatory [7,15], inhibitory [10,14] and or no significant role [18] being reported, again most likely reflecting differences in the experimental conditions.

In the present study, we have investigated platelet activation in mice deficient in the two novel isoforms, PKCθ and PKCε. Although mice deficient in multiple classical isoforms have been described [23], this is the first report of mice lacking two novel isoforms. Mice deficient in PKCθ and PKCε show a marked reduction in aggregation on collagen at arteriolar shear and exhibit a significant increase in tail bleeding *in vivo* relative to wild-type (WT) mice or mice deficient in one novel isoform. These results reveal a combined positive role of PKCε and PKCθ in supporting platelet activation.

## Materials and methods

PKCθ^−/−^/ε^−/−^ mice were bred from PKCθ^−/−^/ε^+/−^ parents on a B6 background and results compared with age-matched wild-type background C57/BL6 and litter-matched PKCθ^−/−^/ε^+/+^ controls. PKCε^−/−^ mouse platelets were compared with wild-type littermate C57/BL6 controls. Animals were bred and blood removed under an approved Home Office Licence (Ref: PPL 30/2721). P-PACK (D-Phe-Pro-Arg-chloromethylketone, HCl) was from Merck Biosciences Ltd (Nottingham, UK). Actin antibody was from Santa Cruz Biotechnology, Inc. (Santa Cruz, CA, USA). PKC antibodies were from BD Biosciences (Oxford, UK), except for PKCα, which was purchased from Cell Signalling Technology (Beverly, MA, USA). Other reagents were from Sigma (Poole, UK) or as previously described [6].

### Washed platelet preparation

Mouse blood was drawn either by cardiac puncture or from the vena cavae of terminally CO_2_-narcosed mice, anesthetized with gaseous isofluorane. Blood was taken into 100 μL ACD and 200 μL modified Tyrodes-Hepes buffer (138 mm NaCl, 2.7 mm KCl, 1 mm MgCl_2_, 3 mm NaH_2_PO_4_, 5 mm glucose and 10 mm HEPES) pH 7.3 and centrifuged at 200 *g* for 6 min; separation from PRP was by spinning at 1000 *g* for 6 min. Platelets were adjusted to ensure a count of at least 10^8^ platelets mL^−1^. Human platelets were isolated as previously described [6] with ethical approval from the Oxford Research Ethics Council (reference number 08/H0605/123).

### Quantification of the PKC isoform levels

The PKC isoforms were quantified using washed platelet samples from three wild-type mice and five human donors using antibodies specific for each isoform. The relative expression of levels of each PKC isoform was determined using quantitative western blotting [24–26]. A range of known concentrations of reference samples, GST-tagged forms of each human PKC isoform (purchased from Enzo Life Sciences, Exeter, UK), were subjected to western blotting and bands were quantified using ECL in combination with the Biorad GelDoc system. Corresponding recognition regions are at least 97% conserved between human and mouse isoforms. Expression levels were calculated by comparing the level of PKC isoforms present in platelet samples with those of the reference samples. The level of expression of PKC isoform per platelet was calculated both as the number of molecules per platelet and as a concentration.

### Aggregometry and ATP release

Aggregation and ATP secretion were monitored following stimulation by the required agonist as previously described using washed platelets [6].

### Flow cytometry

Expression of cell surface glycoproteins was measured by flow cytometry [6].

### Spreading on fibrinogen

Washed platelets (2 × 10^7^ mL^−1^) were exposed to fibrinogen-coated coverslips (100 μg mL^−1^) and adherent platelets were imaged using phase contrast microscopy [27].

### Aggregate formation on collagen under shear

Anticoagulated (heparin [5 IU mL^−1^] and PPACK [40 μm]), whole blood was perfused through collagen-coated capillaries at a shear rate of 1000 s^−1^. Thrombus formation was imaged using phase-contrast microscopy and expressed as the percentage of surface area covered by platelets. Capillary contents were lysed and levels of adherent platelets assessed by western blotting for actin [6].

### Tail bleeding

Tail bleeding experiments were performed on 20–35 g male and female mice, anesthetized with isofluorane and injected with buprenorphine intraperitoneally. The terminal 3 mm of tail was removed using a sharp razor blade and blood collected. Mice were allowed to bleed until they lost either 15% blood volume or for a maximum of 20 min. Data are presented as ratio of amount of blood loss (mg)/mouse weight (g) and rate of blood loss (mg min^−1^).

### Statistical analysis

For all results *n* ≥ 3 for WT, PKCθ^−/−^ and PKCθ^−/−^/ε^−/−^mice. Statistical analyses were carried out on data using unpaired, two-tailed Student’s *t*-test, and *P* < 0.05 was considered statistically significant. Values are expressed as mean ± SEM.

## Results

### PKCθ^−/−^/ε^−/−^ mouse platelets exhibit normal expression of the other PKC isoforms

Quantification of the levels of PKC isoforms in both human and mouse platelets revealed that expression varies over more than two orders of magnitude. PKCθ is the most highly expressed isoform in both species, even though its role has proven difficult to define. In comparison, the level of PKCε expression in mouse platelets is < 5% of that of PKCθ (Fig. S1). The level of PKCε in human platelets, however, is unclear as we were not able to detect expression using commercially available antibodies, although a recent study using an in-house antibody has reported robust expression [8].

**Figure 1:**
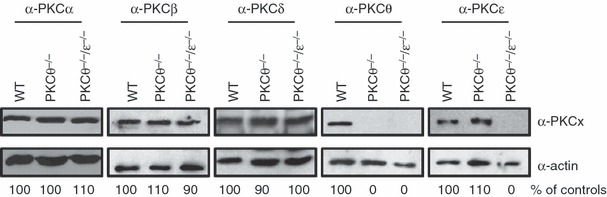
PKCθ^−/−^/ε^−/−^ platelets express normal levels of other PKC isoforms. Equal numbers of washed platelets from WT, PKCθ^−/−^ or PKCθ^−/−^/ε^−/−^ mice were subjected to SDS-PAGE and the expression levels of PKCα, β, δ, θ and ε determined by western blot using anti-sera specific to the individual isoforms of PKC. Actin was used as a loading control and the levels of expression quantified relative to WT. Representative images shown, *n* = 3.

To determine whether any functional redundancy exists between the novel isoforms PKCθ and PKCε, mice deficient in both isoforms were bred and their platelet activity monitored in comparison to PKCθ^−/−^, PKCε^−/−^ and wild-type controls. Mice deficient in both PKCθ and PKCε were indistinguishable from littermate controls for up to 30 weeks and had similar platelet counts and platelet size (data not shown). The expression of the major PKC isoforms was compared in WT, PKCθ^−/−^ and PKCθ^−/−^/ε^−/−^ washed platelet lysates (Fig. 1). As expected, no expression of PKCθ, and neither PKCθ or PKCε, could be detected in platelets purified from PKCθ^−/−^ and PKCθ^−/−^/ε^−/−^ mice, respectively. There was also no significant changes in expression of other PKC isoforms in PKCθ^−/−^/ε^−/−^ platelets relative to PKCθ^−/−^ or WT platelets (Fig. 1). We have also previously reported that expression of other PKC isoforms is not altered in mice deficient solely in PKCε [6]. Expression of GPVI, GPIb and αIIbβ3 were also similar in double-deficient platelets to those in controls (Fig. S2). Similar observations have been reported for the single nulls (Fig. S2) [6,7,14,15,18]. This indicates that any functional differences between the PKCθ^−/−^/PKCε^−/−^ platelets relative to PKCθ^−/−^ and PKCε^−/−^ or WT platelets are not due to altered expression of surface receptors or other PKC isoforms.

### Distinct roles for PKCθ and PKCε in platelet activation

**Figure 2:**
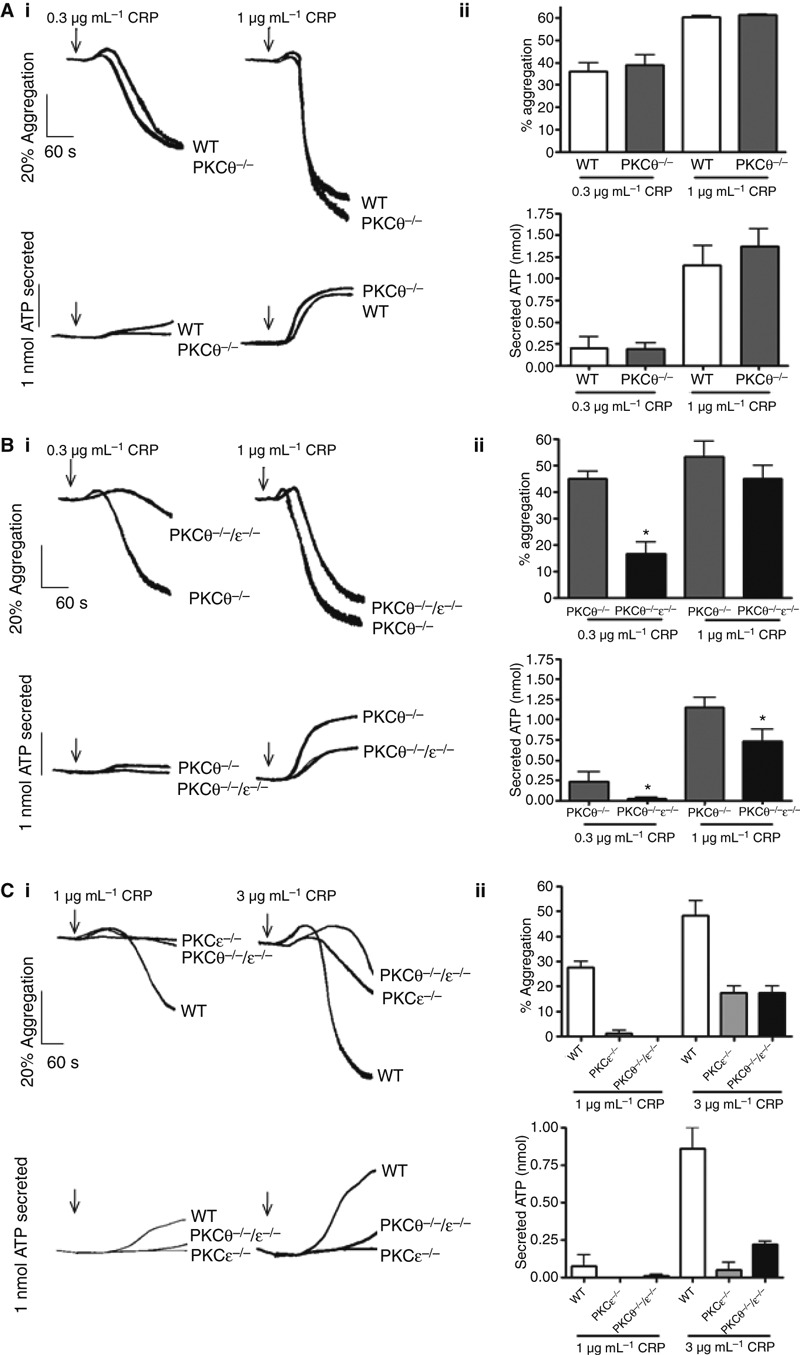
The role of PKCθ and PKCε in GPVI-induced platelet activation. Mouse washed platelets from (A) PKCθ^−/−^ mice or wild-type littermate controls (WT) and (B) PKCθ^−/−^/ε^−/−^ mice or PKCθ^−/−^ littermate controls were stimulated with low (0.3 μg mL^−1^) or just maximal (1 μg mL^−1^) CRP. (C) Mouse washed platelets from PKCθ^−/−^/ε^−/−^, PKCε^−/−^ or wild-type (WT) mice were stimulated with low (1 μg mL^−1^) or high (3 μg mL^−1^) CRP [CRP potency is batch dependent, and so concentrations can vary to induce the same response, therefore although they induce similar submaximal and near maximal responses, the concentrations used in (C) are different to those in (A) and (B)]. Aggregation was measured by optical aggregometry. Dense granule secretion was measured by monitoring ATP secretion using luminometry. (i) Traces representative of *n* = 3 are shown. (ii) Results are average + SEM for *n* = 3 (A and B), *n* ≥ 2 (C). *P < 0.05 in comparison to controls.

We have previously reported a mild defect in aggregation and dense granule secretion in PKCε^−/−^ mouse platelets to the GPVI-specific agonist collagen-related-peptide (CRP) [6]. In contrast, we found no significant difference in aggregation or dense granule secretion in PKCθ^−/−^ mouse platelets relative to controls in response to concentrations of CRP that induce partial and full aggregation (Fig. 2A). Washed platelets from PKCθ^−/−^/ε^−/−^ mice exhibit a similar delay in onset and reduction of aggregation and ATP secretion in response to low and high concentrations of CRP as that previously reported in PKCε^−/−^ mouse platelets (Fig. 2B) [8]). Direct comparison of PKCθ^−/−^/ε^−/−^ and PKCε^−/−^ mouse platelets confirmed a similar defect in CRP-induced responses (Fig. 2C). Thus, PKCθ does not play a critical role in GPVI signaling even in the absence of the novel isoform PKCε. No significant difference was observed in the rate or extent of aggregation or dense granule secretion in PKCθ^−/−^ or PKCθ^−/−^/ε^−/−^ platelets relative to WT mice in response to threshold concentrations of thrombin (Fig. S3).

**Figure 3:**
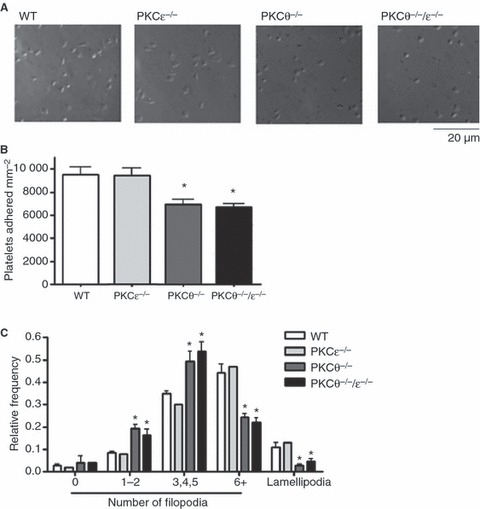
Adhesion to fibrinogen of PKCθ^−/−^/ε^−/−^ mouse platelets. Washed platelets from WT, PKCε^−/−^, PKCθ^−/−^or PKCθ^−/−^/ε^−/−^ mice were exposed to fibrinogen-coated coverslips. (A) Representative phase-contrast images after 4 min. Images were taken under oil immersion. Original magnification, ×63. (B) Number of platelets adhered per mm^2^, calculated by counting the number of cells adhered in three separate images per mouse using Image J analysis. (C) Filopodia number was counted for each visible platelet and the number of platelets with none, few (1–2), some (3–5) or many (6+) filopodia were expressed as relative frequency (proportion of the total number of platelets; more than 100 platelets were counted for each condition). Results are mean + SEM. *P < 0.05 in comparison to WT controls.

**Figure 4:**
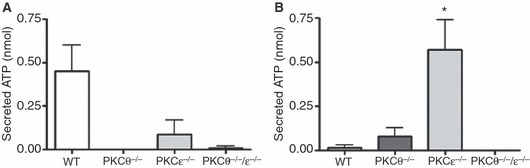
ADP induced dense granule secretion in PKCθ^−/−^/ε^−/−^, PKCε^−/−^ and PKCθ^−/−^ platelets. Mouse PRP from PKCθ^−/−^/ε^−/^, PKCε^−/−^, PKCθ^−/−^ or wild-type (WT) mice were stimulated with 100 μm ADP. Dense granule secretion was measured by monitoring ATP secretion using luminometry. Results are average ± SEM for *n* = 3. *P < 0.05 in comparison to WT controls.

As we have previously shown [6], adhesion and filopodial generation on fibrinogen are not altered in the absence of PKCε (Fig. 3). In contrast, a reduction in adhesion and filopodia generation on a fibrinogen-coated surface was observed in PKCθ^−/−^ platelets in agreement with earlier studies [14,18]. A similar defect was also observed in PKCθ^−/−^/ε^−/−^ platelets (Fig. 4), which was indistinguishable from that seen in PKCθ^−/−^ platelets. Thus, PKCε does not play a critical role in fibrinogen signaling even in the absence of the novel isoform PKCθ.

Platelet activation is reinforced by the feedback agonists ADP and thromboxane A_2_. Aggregation induced by a low concentration of the thromboxane agonist mimetic, U46619, was not altered in platelets deficient in either PKCθ or PKCε or in the absence of both novel isoforms (data not shown). We and others have previously reported that secretion in response to ADP is potentiated in the absence of PKCε, although this did not translate into a change in aggregation [6,8,20]. This result was confirmed in the present study, although interestingly potentiation was not observed in the absence of both PKCε and PKCθ (Fig. 4). This highlights a positive role for PKCθ in the regulation of ADP-induced dense granule secretion, which opposes that of PKCε.

These results suggest isoform-specific rather than redundant roles for the two novel PKC isoforms, PKCθ and PKCε, in supporting platelet aggregation, secretion and adhesion and filopodial generation.

### PKCθ^−/−^/ε^−/−^ platelets show reduced aggregation under shear and mice show bleeding defects

**Figure 5:**
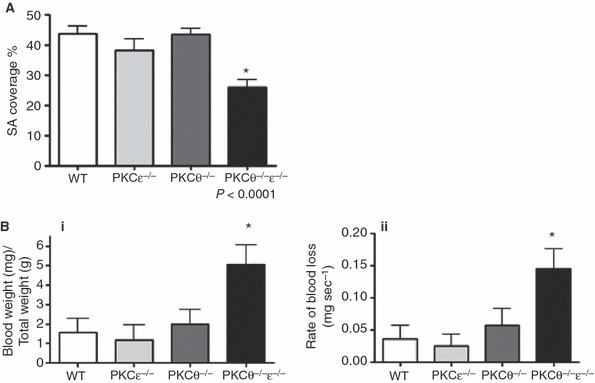
Thrombus formation in PKCθ^−/−^/ε^−/−^ mice. (A) Heparin/PPACK-anticoagulated blood from WT, PKCε^−/−^, PKCθ^−/−^ or PKCθ^−/−^/ε^−/−^ mice was passed over collagen (shear rate 1000 s^−1^). Surface area coverage with thrombi was calculated using three separate images per mouse, mean ± SEM. (B) Tail bleeding as determined by blood lost in 20 min following removal of the terminal 3 mm of the tail; data presented as (i) a ratio of mouse weight and (ii) rate of blood loss (*n* = 10 for WT, six for PKCε^−/−^, 10 for PKCθ^−/−^, and six for PKCθ^−/−^/ε^−/−^). *P < 0.05 in comparison to WT controls.

Our observations suggest isoform-specific rather than redundant roles for PKCθ and PKCε. To determine whether loss of both isoforms had a cumulative effect on platelet function, platelet aggregation and thrombus formation under arteriolar flow rates were investigated by flowing whole blood over immobilized collagen at an arteriolar shear rate of 1000 s^−1^. As we have previously shown, PKCε^−/−^ platelets show no significant difference in aggregate and thrombus formation under these conditions (Fig. 5A) [6]. There was also no significant difference in aggregation on collagen in PKCθ^−/−^ blood under these conditions (Fig. 5A). In contrast, a marked reduction in platelet aggregation on collagen was observed at a shear rate of 1000 s^−1^ in PKCθ^−/−^/PKCε^−/−^ platelets (Fig. 5A), with platelets forming small unstable aggregates. This result demonstrates that at high shear platelet aggregation on collagen is regulated by the combined action of PKCθ and PKCε.

We further investigated whether PKCθ and PKCε are required for hemostasis *in vivo* using a tail bleeding assay. There was no significant increase in bleeding times in the single isoform null mice in comparison to WT controls. In contrast, there was a marked increase in blood lost and time to occlusion in the double-deficient mice (Fig. 5B). This reveals that the two novel isoforms also work in combination to support hemostasis.

## Discussion

It has been proposed that the classical isoforms PKCα and PKCβ play the dominant positive roles in the regulation of platelet activation and thrombus formation, whilst the novel isoforms are thought to play comparatively minor or in some cases negative regulatory roles [9,10]. In the present study, however, we show that the combined loss of the novel isoforms PKCε and PKCθ results in a marked defect in aggregation under flow conditions and a marked increase in tail bleeding, revealing a combined net positive role for the two novel isoforms in hemostasis, which may reflect their individual roles in platelet activation by collagen and fibrinogen, respectively.

PKCθ is the most highly expressed PKC isoform in both human and mouse platelets. Surprisingly, the high level expression of PKCθ is not associated with a major change in GPVI and PAR4 receptor signaling in mouse platelets [7,10,14,15,18,21,22], a result that has been confirmed in the present study. Differences with regard to PKCθ function are most likely due to minor changes in experimental design and the relatively mild role of the novel PKC isoform in platelet activation downstream of GPVI and PAR. On the other hand, PKCθ regulates adhesion and filopodia formation on fibrinogen [14,18], although this is not associated with altered aggregation on collagen at an arteriolar rate of shear (present study) or to a change in tail bleeding time [18]. In comparison, PKCε is expressed at < 5% of the level of PKCθ in mouse platelets and the variable reports of its presence in human platelets are consistent with a low level expression (see Introduction). We have shown that PKCε plays a key role in supporting platelet activation by GPVI [6], as well as secretion (but not aggregation) by ADP in mouse platelets [20].

The use of mice deficient in both PKCε and PKCθ enables the net contribution of the two isoforms to be studied. The *in vitro* studies reveal that the isoform-specific functions of the two novel PKCs are carried over to the double-deficient platelets, with the only departure being the loss of the increased secretion to ADP in the PKCε-null platelets, suggesting that PKCθ opposes this response. Nevertheless, despite the relatively minor phenotype in response to individual agonists, the present study demonstrates a marked reduction in aggregation on collagen under arteriolar flow rates and significant increase in tail bleeding in the combined absence of PKCθ and PKCε. It is already known that the two classical isoforms of PKC, PKCα and PKCβ, play a major role in supporting platelet activation under static and flow conditions [10,12]. The observation of a significant defect in aggregation on collagen at an arteriolar rate of shear and in hemostasis (tail bleeding assay) in the double-deficient mice demonstrates that, in combination, PKCε and PKCθ also contribute to activation as a consequence of distinct roles in platelet activation by GPVI and integrin αIIbβ3, respectively [10,12]. Thus both classical and novel PKC isoforms are required for hemostasis in the arteriolar system.

## Addendum

A. J. Unsworth is supported by a British Heart Foundation (BHF) PhD studentship, L. Navarro-Nunez by the Spanish Ministry of Education (EX2009-0242), B. A. Finney by the Wellcome Trust, and S. Severin by the BHF. S. P. Watson holds a BHF chair. This work was supported by the British Heart Foundation Grant Number FS/08/010/24527. A. J. Unsworth, L. Navarro-Nunez, B. A. Finney and S. Severin performed the research, and analyzed and interpreted data. S. P. Watson and C. J. Pears designed the research and interpreted data. A. J. Unsworth, S. P. Watson and C. J. Pears wrote the manuscript.

## Supporting Information

Figure S1 Quantification of PKC isoforms in human and mouse platelets.

Figure S2 PKCθ^−/−^/^−/−^ platelets express normal levels of major cell surface receptors.

Figure S3 The role of PKCθ andPKC in thrombin induced platelet activation *invitro*.
