# Supplementary figures and images for: Protein kinase Cε and protein kinase Cθ double-deficient mice have a bleeding diathesis
Source: J Thromb Haemost. 2012 Sep 9;10(9):1887–94. doi: 10.1111/j.1538-7836.2012.04857.x (PMC3532618; doi:10.1111/j.1538-7836.2012.04857.x)

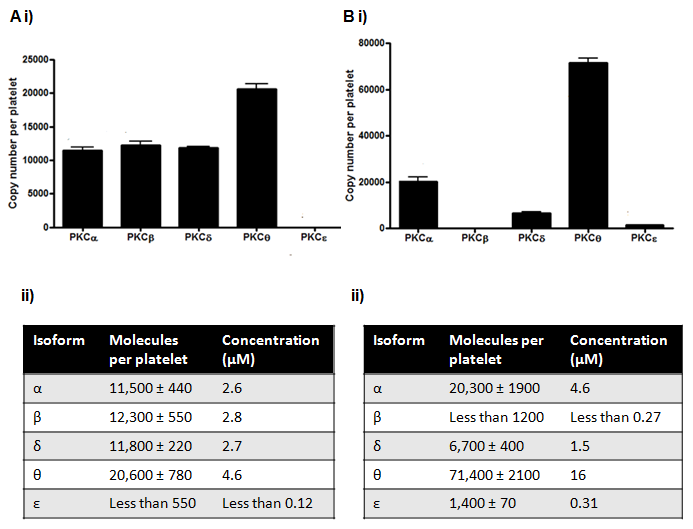

Supplement: Figure S1 — Quantification of PKC isoforms in human and mouse platelets. [file jth0010-1887-SD1.tif]

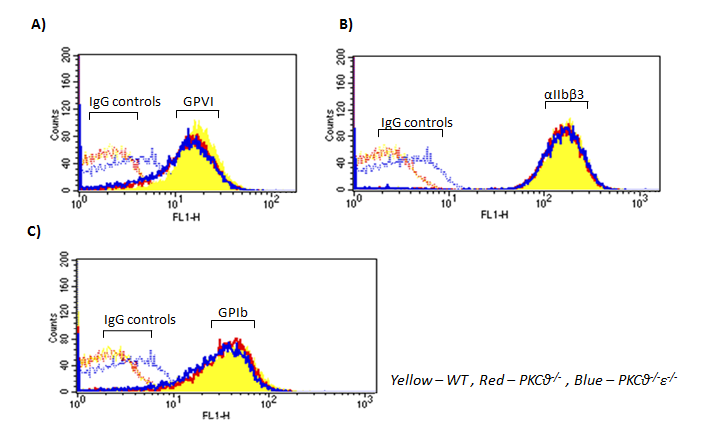

Supplement: Figure S2 [file jth0010-1887-SD2.tif]

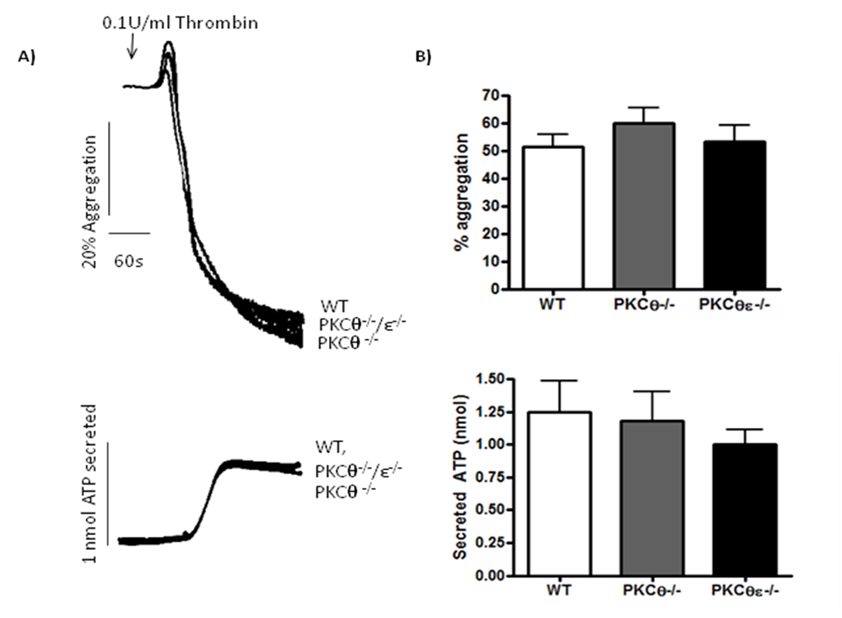

Supplement: Figure S3 [file jth0010-1887-SD3.tif]
